# Hypobaric Packaging Prolongs the Shelf Life of Refrigerated Black Truffles (*Tuber melanosporum*)

**DOI:** 10.3390/molecules25173837

**Published:** 2020-08-24

**Authors:** Sara Savini, Edoardo Longo, Andrea Servili, Sergio Murolo, Massimo Mozzon, Gianfranco Romanazzi, Emanuele Boselli

**Affiliations:** 1 Chemical Safety Department, Stazione Sperimentale per l’Industria delle Conserve Alimentari, Via F. Tanara, 31/A, 43100 Parma, Italy; sr.savini@gmail.com; 2 Faculty of Science and Technology, Free University of Bolzano, Piazza Università, 5, 39100 Bolzano, Italy; edoardo.longo@unibz.it; 3 Department of Agricultural, Food, and Environmental Sciences, Marche Polytechnic University, Via Brecce Bianche, 60131 Ancona, Italy; serviliandrea@libero.it (A.S.); s.murolo@univpm.it (S.M.); m.mozzon@univpm.it (M.M.)

**Keywords:** firmness, microbial epiphytic population, modified atmosphere packaging, volatile compounds, sensory evaluation, shelf life extension

## Abstract

Black truffle (*Tuber melanosporum* Vitt.) is a fine agro-food product known for its unique aroma and very limited shelf life (maximum of 5–7 days, room temperature). Hypobaric packaging at 30 kPa, a mix of 1% O_2_/99% N_2_, and 40% CO_2_/60% N_2_ were studied to prolong the shelf life of black truffle at 4 °C in sealed polypropylene vessels, compared to normal atmosphere. Epiphytic microbial population, firmness, weight loss, CO_2_ formation, and sensory properties were monitored weekly up to 35 days of storage and were related to the volatile profile. Principal components analysis revealed good correlation between the storage time and the decrease of firmness, and the increase of the microbial count and CO_2_ production. Only truffles stored under hypobaric conditions showed an acceptable quality after 14 days storage. Hypobaric packaging is a cheap strategy to prevent the swelling of vessels caused by respiration and can reduce the deviation from the high-quality level of the fresh product from one to at least two weeks.

## 1. Introduction

Truffles are underground tubers living in symbiosis with the roots of host plants [1]. Black truffle (*Tuber melanosporum* Vitt.) is a mushroom known for its unique and characteristic aroma [2], which is exploited in gastronomic preparations. Black truffles are also a valuable and expensive food specialty (up to 600 Euro/kg in Italy, data reported from http://tartufo.acqualagna.com/, accessed on April, 2020) and the demand in the food market is steadily increasing [3,4,5,6,7]. However, a brief harvest period (January to mid-March in the northern hemisphere) and a short shelf life pose a limitation in front of a growing market’s demand [8,9,10]. In the last decade, novel approaches for prolonging the shelf life of fresh truffles have been devised in order to provide more efficient export with fast shipments to far-off markets [11,12,13]. Mencarelli et al. [8] showed that an anoxic atmosphere with 60% CO_2_ and 40% N_2_ may keep the strong typical odor of *Tuber estivum* and can better reduce the superficial growth of molds compared to samples kept in low oxygen, which, in addition, did not prevent the weight loss. Rivera et al. [11] proposed the use of modified atmosphere packaging (MAP) to preserve the sensory and microbiological quality of truffles. The same authors also reported that a high concentration of CO_2_ preserved the quality characteristics of truffles.

Hypobaric storage is an effective technique for extending the shelf life of fresh fruits [14,15]. So far, hypobaric conditions were tested directly on truffles only very recently by the same research group [13,16], who correlated the antioxidant activity of an ethanolic extract of black truffle samples with its minor polar compounds and volatile profile.

In the present work, the epiphytic microbial population, firmness, weight loss, and gas composition of the sealed packages were correlated with the sensory properties (including texture) and the volatile profile in samples of *T. melanosporum*. The effects of hypobaric treatment and other two different types of modified atmosphere packaging (MAP) on those variables were monitored during 35 days of storage of fresh black truffles at 4 °C in sealed polypropylene vessels and compared with vessels sealed under normal atmospheric conditions. The focus of this paper was not the discussion of the physiological aspects of post-harvest of truffle, since previous literature has already tackled these aspects as already reported in this introduction, but to propose to researchers and truffle producers guidelines for a cheap and effective extension of the shelf life of refrigerated truffles in a proper package.

## 2. Results

The shape and the color of fruit bodies were typical of black truffles (*T. melanosporum*) and were characterized by a rounded regular shape, which is usually common to cultivated truffles (by contrast, wild truffles found in natural beds are usually more irregular). The time intervals chosen in this experimentation were surely longer than the effective shelf life of the truffle because of the need to reach the moment of complete degradation of the product also in the best possible conservation conditions.

### 2.1. Weight Loss, Firmness, Gas Composition, and Volatile Profile

The WL data of all MAP at different storage times are reported in Table 1, as well as the area percentage of each volatile compound, the epiphytic microbial population, and truffle firmness (FIRM). The WL increased gradually in all MAP conditions. After 35 days storage, the ON and CN samples showed a lower WL than C and V. The values of firmness ranged from 2.2 to 3.8 g. The very low range of difference related to these variables can be ascribed to the sealed vessels, which prevent the humidity transfer during the storage, unlike what happened with microperforated films [11].

**Figure 1:**
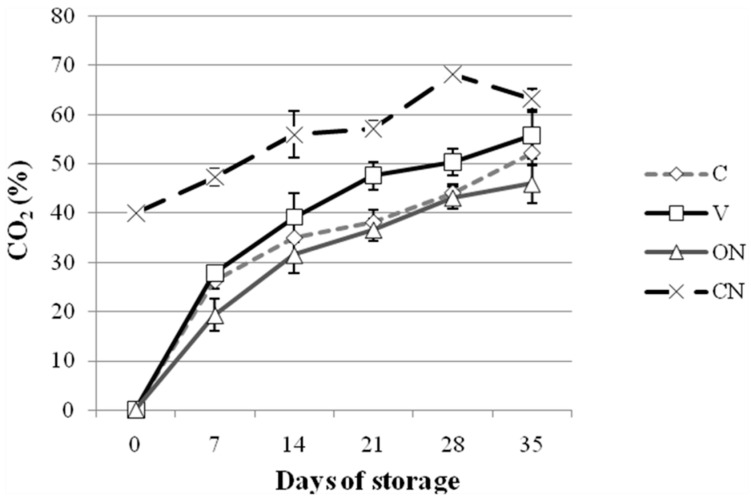
Evolution of the CO_2_ composition of *Tuber melanosporum* in four different MAP: modified atmosphere packaging. C: air atmosphere; CN: mix of 40% CO_2_/60% N_2_; ON: mix of 1% O_2_/99% N_2_; V: hypobaric packaging at 30 kPa. Bars indicate the standard deviation.

The change of the CO_2_ composition inside the vessels is shown in Figure 1.

Oxygen was completely consumed after 7 days in all the vessels, independently of the initial concentration (data not shown). Instead, carbon dioxide constantly increased in all the vessels. This trend is typically due to the high respiration rate of fresh *T. melanosporum* [11].

The SPME-GC/MS characterization of the truffles was performed with the aim to compare the different volatile fingerprints in the relationship with the different storage conditions and time, and thus not for the absolute quantitative determination of the volatile compounds. The internal standard was added to the samples to comply with the cited analytical protocol [13] but was not used to determine the absolute concentration of the volatile components.

**Figure 2:**
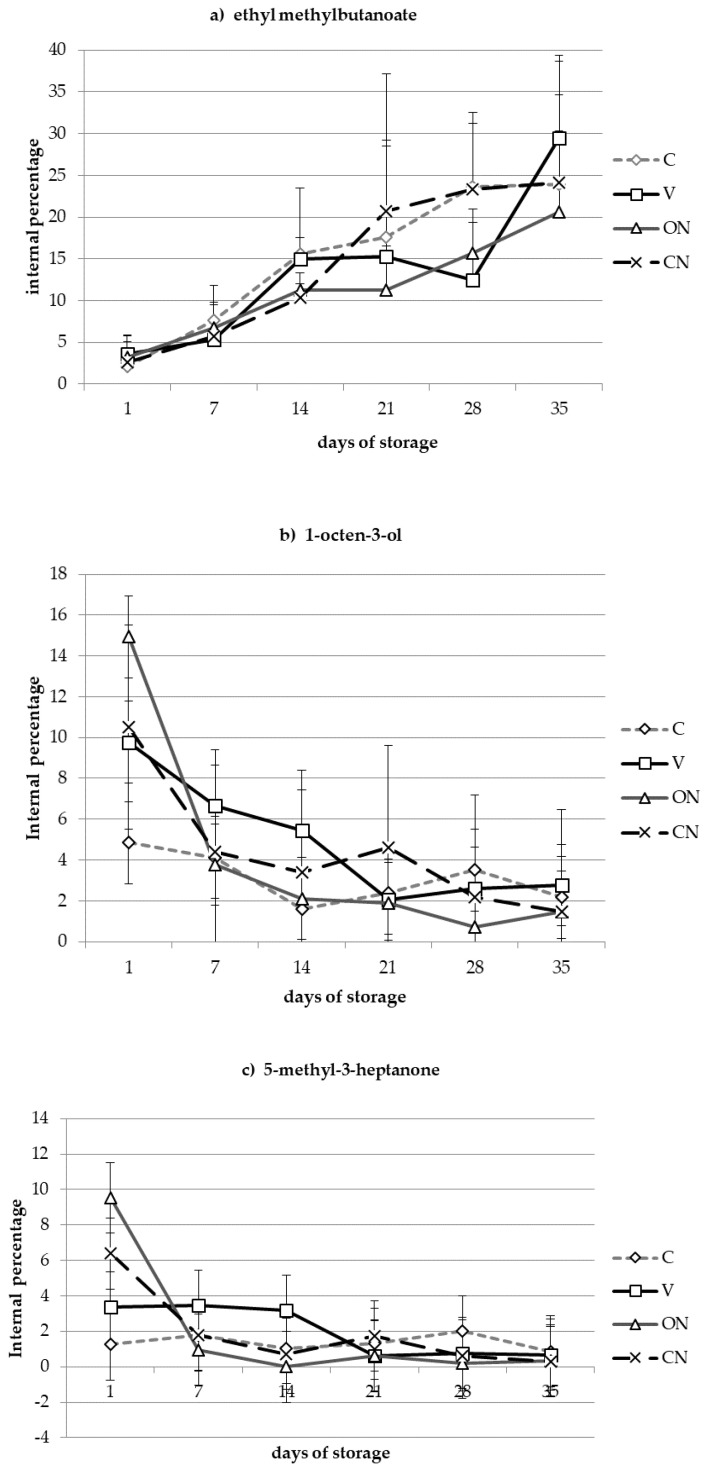
Trend of ethyl methylbutanoate (compound no. **2** in Table 1) (**a**); 1-octen-3-ol (no. 5) (**b**); and 5-methyl-3-eptanone (no. 6) (**c**) during the storage. C: atmospheric pressure (control), V: hypobaric packaging at 30 kPa, ON: 1% O_2_/99% N_2_, CN: 40% CO_2_/60% N_2_. Bars indicate the standard deviation.

The 12 main volatile compounds identified in all the samples (Table 2) had a carbon number distribution between C6 and C8; their relative percentage is reported in Table 1. They were aldehydes, ketones, esters, alcohols, anisole, and two of its derivatives. Three volatile compounds can be considered chemical markers for the refrigerated storage of raw black truffles: Ethyl methylbutanoate is an ester produced upon fermentation of food products [25] or can be typical of fresh fruits [26]. Its formation was directly correlated with the storage in all four packaging conditions (Figure 2a), thus it was related to the decay of the raw black truffles.

Conversely, 1-octen-3-ol (Figure 2b) and 5-methyl-3-heptanone (Figure 2c) were native volatile components of the truffle [18,27,28,29], and decreased during the storage in all treatments. Most of the variation occurred during the first week of storage, no matter the packaging composition. The trend of the other volatile compounds was very changeable and did not significantly correlate with package conditions.

The trends reported in Figure 2 were confirmed by two-way ANOVA, which was applied on the set of the volatile compounds to find significant differences between different atmospheres, times (weeks), and their interaction. Ethyl methylbutanoate and 1-octen-3-ol showed very significant differences (*p* < 0.001) for time only, and 5-methyl-3-heptanone showed a significant interaction between time and atmosphere. Tukey’s HSD (*honestly significant difference*) test was applied for family-wise comparisons: It showed that the compounds showing significant differences for time only had such differences between all atmospheres at week 0 and all later times. Regarding the significance of the interaction, 5-methyl-3-heptanone showed that ON and CN at week 0 differed from all later times and from week 2–5, respectively, whereas A0 and S0 samples differed only from ON0 (no significant interaction between any ON and CN sample).

### 2.2. Microbial Epiphytic Community

The microbial epiphytic population is composed by yeasts and bacteria. Microflora for sure were affected by the treatments, but our investigation aimed to see if there was a change on the amount of propagules and not to investigate in the specific taxa.

Significant differences (*p* < 0.05) were found using two-way ANOVA and a post-hoc HSD test for time but not for the atmosphere used. Week-0 and week-1 differed significantly from week-2, 4, and 5 but not between each other. Week-3 differed also from Week-4 but not from Week-5. This can be amounted to the non-linear increase observed, especially for CN samples. The lowest values were recorded for CN treatment (3.5 × 10^6^ CFU/g), while the highest was registered in the control (8.9 × 10^6^ CFU/g). The treatments V and ON showed intermediate values of the epiphytic microbial population (5.9 × 10^6^ and 6.1 × 10^6^ CFU/g, respectively).

### 2.3. Principal Component Analysis

**Figure 3:**
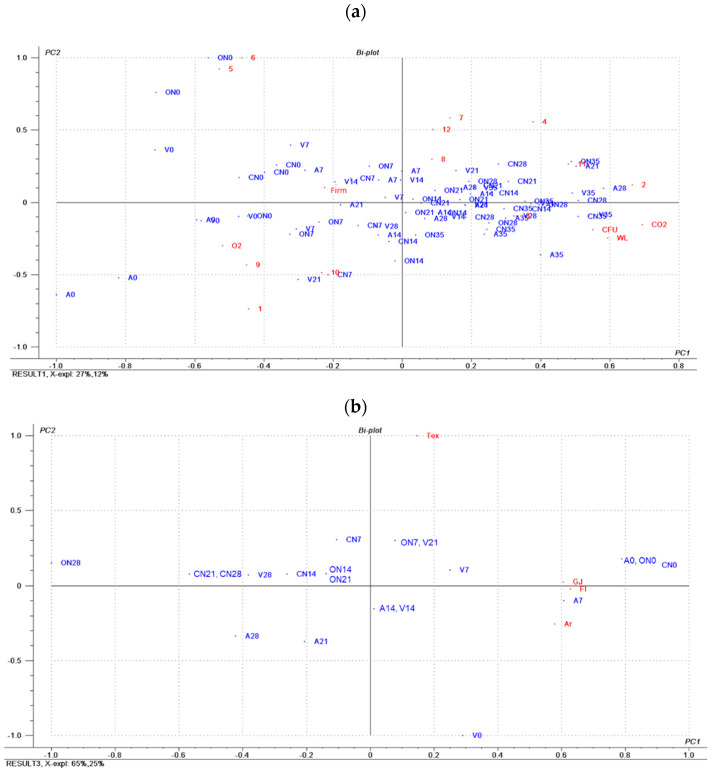
(**a**), Bi-plot of the principal component analysis (PC1 vs. PC2) performed using the normalized volatile compounds profile, physical, and microbial variables. A: atmospheric pressure, control; CN: 40% CO_2_/60% N_2_; ON: 1% O_2_/99% N_2_; V: hypobaric packaging at 30 kPa. 0, 7, 14, 28, and 35 days of storage. 1, hexanal; 2, ethyl methylbutanoate; 3, heptanal; 4, methoxybenzene; 5, 1-octen-3-ol; 6, 5-methyl-3-heptanone; 7, octanal; 8, 1-methoxy-3-methylbenzene; 9, 5-Ethylcyclopent-1-enecarboxaldehyde; 10, octenal; 11, 2-nonen-1-ol; 12, 1,2-dimethoxybenzene. FIRM, instrumental penetrometric determination (g); O_2_ and CO_2_, oxygen and CO_2_ measured % of the head space. (**b**), PCA Bi-plot of the mean sensory scores (PC1 vs. PC2) across the samples. Tex = texture; GJ = global judgement; Fl = flavor; Ar = aroma.

PCA was carried out to get an overview of potential effects on the volatile compounds caused by the specific MAP conditions and to point out potential clustering between samples (Figure 3a). PCA was conducted on 72 samples (truffles in different times of storage in triplicate) across 17 variables (12 volatile compounds, CO_2_, O_2_, WL, CFU, and FIRM). The data set of the truffles was represented in the first two principal components (PC1 × PC2) that explained 39% of the total variance. The score plot of the samples across the variable showed that PC1 depicted a clear separation according to the storage time; in fact, the samples from 0 to 35 days were gradually aligned along the PC1 axis. One of the major contribution variables in the first principal component (depicting the storage time) was the microbial load (CFU), strongly related to the other major contributors, which were the CO_2_ formation and O_2_ loss (by respiration), weight loss, and firmness loss. As expected, firmness (FIRM) was also inversely correlated with WL and CO_2_.

### 2.4. Sensory Analysis

The sensory analysis is commonly considered the most useful tool to rate the quality of commercial fresh truffles. However, to the best of our knowledge, there is no official protocol for the sensory analysis of truffles. The sensory descriptors identified by the sensory evaluation through a preliminary round-table session mediated by the panel leader are reported in Table 3. They were aroma, texture, and flavor. The panel also expressed an overall quality rating (global judgement). An unacceptable score was established for the texture (score 1) and for the global judgement (score 1). The aim of the sensory evaluation was not to establish a limit of acceptability for each sensory attribute, because the truffle samples could be only globally accepted or not.

The typical texture of fresh truffles was not altered in all samples after 7 days of storage. By contrast, differences appeared among the different MAP treatments after 14 days of storage time. The typical aroma and texture of truffles decreased in C samples during 28 days; however, the truffles were considered acceptable by the judges within 28 days of storage. The samples stored for 35 days were impossible to evaluate due to the off-flavors and became unmarketable according to the panel. With the three MAP treatments (V, ON, and CN), the truffles maintained the typical texture until 28 days, but the aroma decreased in the same way in the three packaging conditions (Table 3).

Peculiar sensory attributes, such as butter, chocolate, dried fruit, spicy, yeast, coffee (for flavor) and butter, chocolate, spicy, yeast, coffee, and alcoholic (for aroma), were sporadically perceived by the trained panel only in a few samples and could be related neither to a specific volatile profile nor to MAP conditions.

The data were analyzed with PCA and the results are shown in Figure 3b. The first two principal components (PC1 × PC2) explained 90% of the total variance. The score plot of the samples across the variable showed that PC1 depicted a clear separation according to the storage time, in fact the samples from 0 to 35 days were gradually aligned along the PC1 axis. The samples with the highest sensory impact (mainly time 0 and 7) are those located close to the four sensory variables (Tex, GJ, Fl, Ar). The worst samples, from the sensory point of view, are those located along the negative values of PC1. Thus, according to this model, all the samples stored for 28 days can be considered of low quality, whereas only the samples stored for 21 days packed under hypobaric conditions showed positive values on the PC1 and PC2 axis.

## 3. Materials and Methods

### 3.1. Sampling Procedure

The sampling procedure was the same as described by Savini et al. [13] Briefly, fresh intact samples of *T. melanosporum* were harvested in the hilly areas of the Marche Region (central Italy) by a private company (AcqualagnaTartufi, Acqualagna, PU, Italy). The sampling procedure was immediately carried out in the facility of the supplier under the supervision of the university team. After manual cleaning, the fresh samples of truffle were gently brushed. Then, the truffles were weighed and packed into polypropylene tub-type vessels (140 × 175 × 46 mm) (Tecnowerk Plast, Arsiè, Italy). A thermo-sealing machine mod. Rio 39 from Saccardo (Thiene, Italy) was used to pack the vessels with three different atmosphere compositions and under reduced pressure. The package was sealed with an antifog film from Nutripack (Milan, Italy), consisting of a polyester film of 12 µm adherent to a polypropylene film weldable on PP homopolymer vessels. The composition of the 4 different atmospheres used for the study was the following: Air at atmospheric conditions (C) (control); air, hypobaric packaging at 30 kPa (0.3 bar) (V), 1% O_2_/99% N_2_ (ON), 40% CO_2_/60% N_2_ (CN). All the food-grade gases were provided by Sapio (Monza, Italy). Each sample (sealed vessel) was composed by 5 truffles weighing overall about 100 g. Three replicates (three different vessels) were used for each sampling point. The total number of vessels was 72, that is 12 (4 atmospheres for 3 replicates) samples for each of the 6 sampling times (0, 7, 14, 21, 28, 35 days). All the sealed vessels were weighed and immediately refrigerated and kept at 4 °C, including the control samples. The samples were analyzed at predetermined storage time intervals: 0, 7, 14, 21, 28, and 35 days. At time 0, the samples were analyzed the morning after the packaging procedure. The analyses included the monitoring of the weight loss (WL), gas composition of the sealed vessels, profile of the volatile fraction, microbial epiphytic population, firmness, and sensory analysis. All the results are expressed as mean ± standard deviation of three replicates.

### 3.2. Atmosphere Composition, Weight Loss, and Firmness

The percentage of oxygen and carbon dioxide inside each sealed vessel was determined using a gas sensor PBI by Dansensor (Segrate, Italy). At each time of analysis, the truffles were weighed to determine the weight loss (WL). The firmness of the samples (expressed in g) was evaluated during the storage by using a penetrometer (Fruit Pressure Tester 327, Effegì, Ravenna, Italy) with a 6-mm star head [30].

### 3.3. Volatile Aroma Profile

Solid-phase microextraction-gas chromatography/mass spectrometry (SPME-GC/MS) was applied to analyze the volatile compounds of refrigerated black truffle as reported in a previous work [13]. An aliquot (1.5 g) of fresh sample was finely ground using a mill (IKA M20, IKA-Werke GmbH&Co, Staufen, Germany). The powder was introduced into a 10-mL glass vial closed with a screw cap equipped with an elastomeric septum. The vial was placed in a heating bath at 40 °C for 10 min. Afterwards, an SPME fiber (Divinylbenzene/Carboxen/Polydimethylsiloxane, 1 cm, 50/30 µm) from Supelco/Sigma-Aldrich (Milan, Italy) was introduced and exposed to the headspace of the sample for 15 min. Thermal desorption of the compounds from the fiber took place in the GC injector at 220 °C for 15 min. The GC-MS runs were performed with a Varian 3900 gas chromatograph coupled to a Saturn 2100T (Varian, Walnut Creek, CA, USA) ion trap mass spectrometer. The chromatographic separation was performed on a TG–5MS capillary column (Thermo Scientific, Waltham, MA, USA, 30 m × 0.25 mm I.D., film thickness 0.25 µm). The oven temperature program started at 40 °C was held for 10 min, and then raised by 3 °C/min to 180 °C and to 250 °C at a rate of 15 °C/min. The MS transfer line and ionization chamber temperature were set to 200 °C. The mass spectra were recorded in full scan mode (mass range 31–250 *m*/*z*) with a scan rate of 1 scan/s. The injection was in splitless mode (splitless time 0.3 min) at 220 °C. The data integration was performed automatically using the Varian Workstation software (Varian Inc., Palo Alto, CA, USA). The total ion current (TIC) peaks were expressed as areas vs. retention time and were manually aligned using the MS spectra to monitor the correctness of the alignment. The peaks were assigned using an integrated approach: (a) By comparison with reference mass spectra (NIST/EPA/NIH Mass Spectral Library Version 2.0); (b) possible isomers/analogues were assigned by calculating the retention indexes (RI) on the basis of the elution series of linear alkanes standards. Consequently, non-isothermal linear retention indexes were calculated for the identified compounds from the reference alkane standards retention times. The NIST library was searched for matching the acquired and reference MS spectra and for comparing the measured and theoretical values of RI for the most likely assignments and the most similar stationary phases (as reported in Table 2). Pure standard volatile compounds obtained from Sigma-Aldrich (Milan, Italy) were injected to confirm the identification of the compounds no. **1**, **2**, **3**, **4**, **5**, **6**, **7**, **8**, **12** of Table 2. One-way ANOVA showed no significant differences (*p* < 0.05) in the volatile profiles of three replications of the same sample of black truffle, which were analyzed by SPME-GC/MS.

### 3.4. Microbial Epiphytic Population

To monitor the microbial epiphytic population (sum of yeasts, fungi, and bacteria), the truffles were suspended in 500-mL Erlenmeyer flasks with 500 mL of sterile distilled water, and placed on a rotary shaker set at 200 rpm for 30 min at 20 °C. Washing water aliquots of 100 µL of appropriate dilutions were uniformly distributed in five Petri dishes containing Potato Dextrose Agar (PDA) addition of streptomycin sulphate and ampicillin (250 mg/L each) to isolate yeasts, yeast like-fungi, and filamentous fungi, or 100 mg/L of cycloheximide to isolate bacteria [31]. The plates were incubated at 25 °C under light conditions. After 4–7 days, the colonies of yeasts, yeast-like fungi, filamentous fungi, and bacteria were recorded and transferred to fresh PDA. The total epiphytic microbial population was expressed as colony-forming units per gram of truffle (CFU/g).

### 3.5. Sensory Analysis

A panel of eight trained judges evaluated the sensory properties of the truffles. The sensory evaluation was conducted using a score card, including specific sensory descriptors, which were defined in a preliminary session with the round-table method [32,33]. The sensory descriptors were texture, aroma (butter, chocolate, spicy, yeast, coffee, alcoholic), and flavor (butter, chocolate, dried fruit, spicy, yeast, coffee) with a corresponding rating scale. The perception of aroma was defined by the following scores: 1 = no typical aroma; 3 = moderate typical aroma; 5 = full typical aroma. Successively, the truffles were sliced and judged on a scale, where 1 = no typical flavor; 3 = moderate typical flavor; 5 = full typical flavor. The global judgment was evaluated using a scale from 1 to 5, where 1 = unacceptable and 5 = excellent. The texture was evaluated with a little pressure between the thumb and index. The reference scale was the same used by Rivera et al. [11]: 1 = unacceptable; 3 = soft; 5 = moderately soft; 7 = moderately hard; 9 = hard.

### 3.6. Statistical Analysis

The one-way and two-way ANOVA was performed using the GraphPad Prism 5 software (GraphPad Software Inc., La Jolla, CA, USA). Principal components analysis (PCA) was performed using the Unscrambler software v 9.7 by Camo (Oslo, Sweden) on normalized data (correlation matrix).

## 4. Conclusions

The use of MAP with CO_2_ and N_2_ has been proposed in the past [8] to maintain the sensory and microbiological quality of truffles during refrigerated storage. However, hypobaric packaging has never been considered before by other research groups. Our results showed that hypobaric packaging is a good option for the refrigerated storage of raw black truffles for its low cost and reliable performances. In addition, the presence of reduced pressure minimized the swelling of the pack associated with the CO_2_ production during the storage. This type of packaging may represent a good compromise for ensuring microbial stability, sensory properties, and weight loss up to 14 days of refrigerated storage or longer. Hypobaric packaging delayed the superficial development of molds, avoiding the formation of off-odors and off-tastes, and contributed to maintain the desired hard texture of the black truffles. In conclusion, hypobaric packaging offered the cheapest approach to prolong the shelf life of black truffles from 7 days up to two weeks or longer, keeping a low deviation from the high-quality level of the fresh product. This is very important for small- and medium-sized businesses that export this fine fresh and valuable product to far-off markets. This solution represents also an economic advantage both for the customer and for the producer because the supply towards the final consumer can be done with longer time intervals.

## Figures and Tables

**Table 1:** Characterization of refrigerated black truffles. CFU (colony-forming units related to the sum of yeasts, fungi, and bacteria), FIRM (Instrumental Penetrometric Determination), volatile compounds (from **1** to **12**), WL (weight loss) of fresh *T. melanosporum* initially (T0) and at 7, 14, 21, 28, and 35 days of storage time (T) at 4 °C in different atmosphere conditions.

T	Sample ^1^	CFU/g	FIRM ^2^	1 ^3^	2	3	4	5	6	7	8	9	10	11	12	WL%
T0	C ^2^	3.8 × 10⁵ ^a^	3.7 ^a^	35.1 ^a^	2.1 ^a^	4.6 ^a^	4.6 ^a^	4.8 ^a^	1.2 ^a^	2.1 ^a^	ND	29.5 ^a^	9.2 ^a^	2.9 ^a^	3.8 ^a^	0.0 ^a^
	V	2.6 × 10⁵ ^a^	3.0 ^a^	41.3 ^a^	3.6 ^a^	9.8 ^a^	8.7 ^a^	9.8 ^a,b^	3.3 ^a,c^	1.9 ^a^	2.1 ^a^	4.2 ^b^	3.0 ^b^	2.7 ^a^	9.7 ^a^	1.0 ^a^
	ON	3.4 × 10⁵ ^a^	3.2 ^a^	27.7 ^a^	3.2 ^a^	4.4 ^a^	8.0 ^a^	14.9 ^b^	9.5 ^b,c^	3.5 ^a^	7.1 ^a^	6.8 ^b^	2.7 ^b^	5.5 ^a^	6.7 ^a^	1.0 ^a^
	CN	1.1 × 10^6 a^	3.2 ^a^	39.1 ^a^	2.6 ^a^	3.1 ^a^	5.6 ^a^	10.5 ^a,b^	6.4 ^b,c^	3.8 ^a^	2.2 ^a^	9.2 ^b^	4.9 ^a^	4.0 ^a^	8.5 ^a^	1.3 ^a^
T7	C	1.2 × 10⁵ ^a^	2.8 ^a^	29.6 ^a^	7.6 ^a^	10.0 ^a^	11.8 ^a^	4.1 ^a^	1.8 ^a^	4.1 ^a^	2.3 ^a^	6.0 ^a^	1.9 ^a^	5.9 ^a^	15.0 ^a^	0.7 ^a^
	V	4.8 × 10⁵ ^a^	2.5 ^a^	33.9 ^a^	5.3 ^a^	10.4 ^a^	9.4 ^a^	6.7 ^a^	3.5 ^a^	2.4 ^a,b^	2.1 ^a^	8.3 ^a^	3.5 ^a^	5.2 ^a^	9.5 ^a^	2.0 ^a^
	ON	4.1 × 10⁵ ^a^	3.2 ^a^	38.3 ^a^	6.7 ^a^	7.9 ^a^	8.1 ^a^	3.8 ^a^	1.0 ^a^	1.3 ^b^	13.4 ^a^	5.3 ^a^	1.5 ^a^	3.5 ^a^	9.3 ^a^	1.7 ^a^
	CN	2.2 × 10^6 a^	3.2 ^a^	44.7 ^a^	5.7 ^a^	11.5 ^a^	7.5 ^a^	4.4 ^a^	1.8 ^a^	1.4 ^a,b^	3.9 ^a^	4.4 ^a^	5.2 ^a^	2.7 ^a^	6.7 ^a^	1.7 ^a^
T14	C	2.9 × 10^6 a^	3.7 ^a^	28.0 ^a^	15.6 ^a^	15.8 ^a^	9.7 ^a^	1.6 ^a^	1.1 ^a^	1.9 ^a^	1.7 ^a^	4.2 ^a^	3.8 ^a^	10.0 ^a^	6.7 ^a^	3.0 ^a^
	V	3.6 × 10^6 a^	3.8 ^a^	27.2 ^a^	14.9 ^a^	10.0 ^a^	9.3 ^a^	5.4 ^a^	3.2 ^a^	1.4 ^a^	2.9 ^a^	5.5 ^a^	3.0 ^a^	8.3 ^a^	8.8 ^a^	3.3 ^a^
	ON	5.0 × 10^6 a^	3.2 ^a^	34.8 ^a^	11.3 ^a^	15.4 ^a^	8.3 ^a^	2.1 ^a^	ND	2.8 ^a^	3.2 ^a^	5.7 ^a^	3.3 ^a^	6.3 ^a^	6.7 ^a^	3.4 ^a^
	CN	6.9 × 10^6 a^	2.9 ^a^	28.8 ^a^	10.3 ^a^	16.4 ^a^	10.9 ^a^	3.4 ^a^	0.7 ^a^	2.2 ^a^	5.1 ^a^	5.5 ^a^	2.6 ^a^	7.8 ^a^	6.3 ^a^	3.6 ^a^
T21	C	4.8 × 10^6 a^	3.2 ^a^	29.3 ^a^	17.6 ^a^	10.0 ^a^	11.3 ^a^	2.4 ^a^	1.3 ^a^	2.7 ^a^	1.3 ^a^	1.9 ^a^	0.8 ^a^	8.9 ^a^	12.3 ^a^	4.3 ^a^
	V	1.8 × 10^6 a^	3.6 ^a^	39.6 ^a^	15.3 ^a^	10.3 ^a^	8.1 ^a^	2.1 ^a^	0.6 ^a^	1.9 ^a^	1.1 ^a^	2.8 ^a^	0.8 ^a^	9.8 ^a^	7.4 ^a^	4.6 ^a,b^
	ON	1.1 × 10^6 a^	3.5 ^a^	18.9 ^a^	11.3 ^a^	13.1 ^a^	8.0 ^a^	1.9 ^a^	0.6 ^a^	2.6 ^a^	19.5 ^b^	6.6 ^a^	1.7 ^a^	5.9 ^a^	9.9 ^a^	7.3 ^a,b^
	CN	2.4 × 10^6 a^	3.2 ^a^	21.5 ^a^	20.7 ^a^	14.2 ^a^	10.3 ^a^	4.6 ^a^	1.7 ^a^	2.6 ^a^	3.0 ^a^	4.1 ^a^	1.3 ^a^	6.8 ^a^	9.3 ^a^	3.4 ^a,c^
T28	C	8.9 × 10^6 a^	2.9 ^a^	24.5 ^a^	23.6 ^a^	6.3 ^a^	10.6 ^a^	3.5 ^a^	2.0 ^a^	1.7 ^a^	2.6 ^a^	4.4 ^a^	1.2 ^a^	9.8 ^a^	9.6 ^a^	4.8 ^a^
	V	5.9 × 10^6 a,b^	2.4 ^a^	28.6 ^a^	12.4 ^a^	16.6 ^a^	9.6 ^a^	2.6 ^a^	0.8 ^a^	0.9 ^a,b^	8.6 ^a,b^	3.1 ^a^	1.9 ^a^	4.4 ^a^	10.4 ^a^	3.0 ^a,b^
	ON	6.1 × 10^6 a,b^	2.9 ^a^	16.3 ^a^	15.7 ^a^	6.7 ^a^	9.7 ^a^	0.7 ^a^	0.2 ^a^	2.2 ^a,b,c^	20.0 ^b^	4.9 ^a^	2.3 ^a^	11.7 ^a^	9.6 ^a^	5.8 ^a,b^
	CN	3.5 × 10^6 b^	2.7 ^a^	19.3 ^a^	23.3 ^a^	10.5 ^a^	8.7 ^a^	2.2 ^a^	0.6 ^a^	4.4 ^a,c^	3.5 ^a^	5.5 ^a^	1.4 ^a^	6.4 ^a^	14.2 ^a^	7.9 ^a,c^
T35	C	7.8 × 10^6 a^	2.2 ^a^	29.3 ^a^	23.9 ^a^	6.2 ^a^	9.4 ^a^	2.1 ^a^	0.8 ^a^	1.7 ^a^	5.8 ^a^	3.3 ^a^	6.4 ^a^	5.5 ^a^	5.1 ^a^	9.9 ^a^
	V	3.8 × 10^6 a^	2.5 ^a,b^	15.0 ^a^	29.4 ^a^	10.6 ^a^	7.9 ^a^	2.7 ^a^	0.7 ^a^	3.6 ^a^	3.8 ^a^	3.8 ^a^	1.6 ^a^	12.1 ^a^	8.3 ^a^	9.3 ^a^
	ON	4.1 × 10^6 a^	3.4 ^b^	25.3 ^a^	20.6 ^a^	8.4 ^a^	13.1 ^a^	1.5 ^a^	0.4 ^a^	2.8 ^a^	2.5 ^a^	3.3 ^a^	1.2 ^a^	13.1 ^a^	7.4 ^a^	7.0 ^a^
	CN	4.3 × 10^6 a^	2.4 ^a,b^	22.5 ^a^	24.1 ^a^	11.1 ^a^	9.9 ^a^	1.5 ^a^	0.3 ^a^	2.2 ^a^	1.0 ^a^	3.1 ^a^	4.6 ^a^	9.3 ^a^	10.1 ^a^	6.7 ^a^
Pooled standard deviation		4.5 × 10^6^	1.1	30.7	17.7	10.7	7.0	5.9	4.3	2.8	15.5	11.2	5.6	10.1	9.4	3.6

All data are reported as the mean of three determinations (three packages per control day and batch). Volatile compounds are reported as internal area percentages. Different letters in each column for each storage time represent significant differences (*p* < 0.05). The pooled standard deviation (sd) for each parameter is reported in the last line. ^1^ C: air atmosphere; CN: mix of 40% CO_2_/60% N_2_; ON: mix of 1% O_2_/99% N_2_; V: hypobaric packaging at 30 kPa. ^2^ measured in g. ^3^ Identified aroma compounds (as listed in Table 2): **1**, hexanal; **2**, ethyl methylbutanoate; **3**, heptanal; **4**, methoxybenzene; **5**, 1-octen-3-ol; **6**, 5-methyl-3-heptanone; **7**, octanal; **8**, 1-methoxy-3-methylbenzene; **9**, 5-Ethylcyclopent-1-enecarboxaldehyde; **10**, octenal; **11**, 2-nonen-1-ol; **12**, 1,2-dimethoxybenzene.

**Table 2:** Most representative volatile compounds determined with HS-SPME-GC/MS. All the compounds were identified by mass spectrometric fragmentation, retention time (Rt), and retention index (RI). The compound no. **1**, **2**, **3**, **4**, **5**, **6**, **7**, **8**, **12** were identified by also injecting the standard compounds.

Peak#	Compound Identified	Formula	MW	CAS# ^a^	Rt	RI	Ref. ^b^
**1**	hexanal	C_6_H_12_O	100	66-25-1	6.84	807	[17]
**2**	ethyl methylbutanoate	C_7_H_14_O_2_	130	7452-79-1 ^c^/108-64-5 ^d^	8.78	839 ^c^/844 ^d^	[18]
**3**	heptanal	C_7_H_14_O	114	111-71-7	10.80	906	[17]
**4**	methoxybenzene (alias anisol)	C_7_H_8_O	108	100-66-3	11.40	918	[19]
**5**	1-octen-3-ol	C_8_H_16_O	128	3391-86-4	14.29	985	[20]
**6**	5-methyl-3-heptanone	C_8_H_16_O	128	541-85-5	14.58	954	[21]
**7**	octanal	C_8_H_16_O	128	124-13-0	15.19	1007	[17]
**8**	1-methoxy-3-methylbenzene	C_8_H_10_O	122	100-84-5	15.82	1028	[22]
**9**	5-Ethylcyclopent-1-enecarboxaldehyde	C_8_H_12_O	124	36431-60-4	16.24	1026	[18]
**10**	(*E*)-2-octenal	C_8_H_14_O	126	2548-87-0	17.33	1063	[17]
**11**	2-nonen-1-ol	C_9_H_18_O	142	22104-79-6	19.22	1105	[23]
**12**	1,2-dimethoxybenzene (alias veratrol)	C_8_H_10_O_2_	138	91-16-7	20.83	1143	[24]

^a^ Chemical Abstract Service number; ^b^ Normal alkane RI for a non-polar column with temperature ramp (5% diphenyl, 30m/0.25mm/0.25 µm, He, 40 °C initial oven temperature or similar). ^c^ ethyl 2-methylbutanoate; ^d^ ethyl 3-methylbutanoate.

**Table 3:** Sensory characteristics (texture, aroma, and flavor) of fresh sample of *T. melanosporum* initially (0) and at 7, 14, 21, and 28 days of storage at 4 °C in modified atmosphere. All data are expressed as the mean of three determinations (three truffles per batch evaluated by 7 judges). Sporadically perceived sensory attributes were not reported.

Storage Time (days)	Texture ^1^				Aroma				Flavor				Global Judgement			
	C ^2^	V	ON	CN	C	V	ON	CN	C	V	ON	CN	C	V	ON	CN
0	9.0	4.0	9.0	9.0	4.5	5.0	4.5	5.0	5.0	4.0	5.0	5.0	5.0	4.0	5.0	5.0
7	8.0	8.5	9.0	9.0	5.0	4.0	3.0	3.0	5.0	4.0	4.0	3.0	4.0	4.0	4.0	4.0
14	7.0	7.0	8.0	8.0	3.0	3.0	3.0	3.0	4.0	4.0	3.0	3.0	4.0	4.0	4.0	3.5
21	6.0	9.0	8.0	8.0	3.0	3.0	3.0	3.0	3.0	4.0	3.0	2.0	4.0	4.0	4.0	3.0
28	6.0	8.0	8.0	8.0	2.5	3.0	2.0	3.0	3.0	3.0	2.0	2.0	3.5	3.0	2.0	3.0
Pooled standard deviation	1.30	1.99	0.55	0.55	1.08	0.89	0.89	0.89	1.0	0.45	1.14	1.22	0.55	0.45	1.10	0.84

^1^ Texture evaluated on a 1–9 scale (1 = unacceptable; 3 = soft; 5 = moderately soft; 7 = moderately hard; 9 = hard); Aroma scored on a 1–5 scale (1 = no typical aroma; 3 = moderate aroma; 5 = full typical aroma); Flavor scored on a 1–5 scale (1 = no typical flavor; 3 = moderate flavor; 5 = full typical flavor). Global judgment scored on a 1–5 scale (1 = unacceptable; 3 = moderately acceptable; 5 = excellent). ^2^ C: air atmosphere; CN: mix of 40% CO_2_/60% N_2_; ON: mix of 1% O_2_/99% N_2_; V: hypobaric packaging at 30 kPa.

## Abbreviations

Ar	aroma
C	atmospheric conditions
CAS#	Chemical Abstract Service number
CFU	colony forming units
CN	40% CO_2_/60% N_2_
FIRM	Instrumental Penetrometric Determination
Fl	flavor
GJ	global judgement (sensory)
MAP	modified atmosphere packaging
MW	molecular weight
ON	1% O_2_/99% N_2_
PDA	Potato Dextrose Agar
RI	retention index in gas chromatography
Rt	retention time in gas chromatography
SPME	solid phase microextraction
Tex	texture (sensory)
V	hypobaric packaging with air at 30 kPa (0.3 bar)
WL	weight loss
